# SLAP Trial: Shock Wave Lithotripsy and Mechanical Percussion Therapy Post ESWL for Renal Calculi

**DOI:** 10.1155/2024/7870425

**Published:** 2024-03-26

**Authors:** Nishal Patel, Adrian Roe, Donna Stanton, Jay Roberts, Akshay Kothari

**Affiliations:** ^1^Department of Urology, The Prince Charles Hospital, Brisbane, Queensland, Australia; ^2^Faculty of Medicine, The University of Queensland, Brisbane, Queensland, Australia; ^3^Department of Urology, Royal Brisbane and Women's Hospital, Brisbane, Queensland, Australia

## Abstract

**Methods:**

We conducted a prospective randomised control trial. Included patients were males and females greater than 18 years of age with single or multiple ipsilateral renal calculi of total ≤10 mm on plain X-ray and noncontrast CT KUB. ESWL was performed at a single centre, at supine position under general anaesthesia with maximum 3000 shocks at a rate of 100 shocks per minute. Patients were discharged and randomised to either the control arm or MPI therapy. MPI therapy was self-directed in a home setting for 10 minutes a day, three times per week. Both arms had standard follow-up at 12 weeks with a plain X-ray KUB. Patients in the control group were offered cross over to the MPI arm after 12 weeks if residual stone fragments were detected. Statistical analysis was performed using SPSS software via Chi squared and Fisher's exact tests. Ethical approval was obtained via the Prince Charles Hospital HREC Committee, HREC/2022/QPCH/84961.

**Results:**

70 patients met inclusion criteria and underwent ESWL, and 5 were withdrawn. 33 patients were randomised to the MPI group and 32 to the control group. MPI significantly increased the stone clearance rate anywhere in the kidney (87.9% in the MPI group versus 59.4% in the control group, *p*=0.089), as well as the clearance rate in the lower pole (91.7% in the MPI group versus 63.2% in the control group, *p*=0.022). Delayed percussion did not improve the clearance rate over primary percussion (*p*=0.835).

**Conclusion:**

This study has shown that MPI can be effectively performed in a home setting without the need for medical supervision and results in improved stone clearance rates post ESWL. The main limitations to the study were the use of X-ray over CT during the follow-up and variability in MPI compliance and administration. Further research is warranted into standardising home MPI protocols. This trial is registered with ANZCTR387061.

## 1. Introduction

Renal stone disease represents a heterogenous disease population. The need for treatment is dependent on several factors; stone growth, symptomatology, infection, and patient preference [1]. The diverse range of stone sizes, composition, and location all significantly affect optimal treatment modality [2, 3].

ESWL has long been the mainstay in treatment of small to medium sized renal stones, which is favoured because of its low cost and noninvasive nature compared to endoscopic extraction or percutaneous nephrolithotomy (PCNL). Stones in the lower pole (LP) of the kidney represent a unique subset of cases, characterised by inherent treatment difficulty either in stone access for fragmentation and endoscopic extraction or by retained fragments after ESWL due to impaired drainage [1]. This prompted research into methods to optimise fragment drainage out of the lower pole. One of these methods is the use of mechanical percussion and inversion therapy.

Mechanical percussion therapy aims to create vibration and movement in a treated kidney to increase activity of stone fragments within the calyces in the hope of increasing spontaneous passage into the renal pelvis and down the ureter. Furthermore, inversion of patients aims to counteract the adverse factors of the gravity dependent lower pole and steep infundibulo-pelvic angle by bringing the collecting system beyond the horizontal plane. This theory was built on earlier studies which demonstrated increased passage of stone fragments with certain physical activities, such as riding a roller coaster or sexual intercourse, as well as sleep positioning [4–6].

An early pilot study in the year 2000 demonstrated proof of concept for the use of mechanical percussion and inversion by documenting movement of fragments out of the lower pole in 11 out of 12 patients [7]. A subsequent study randomised 69 patients with residual fragments 3 months after lithotripsy to either mechanical percussion and inversion (MPI) or observation alone. After four weeks of therapy, they offered crossover of the observation group to MPI. They found a significant increase in stone-free rates (SFRs) in the percussion group with 40% SFR compared to only 3% in the observation group (*p* < 0.001) [8]. The effect of MPI was identical whether performed immediately after percussion or after the period of observation. Another study randomised 108 patients to receive percussion, diuresis, and inversion therapy (PDI), commencing 1-2 weeks following ESWL or ESWL alone. At three months, the clearance rates for the PDI group and ESWL alone group were 62.5% and 35.4%, respectively, (*p*=0.006), showing a significant improvement in stone-free rates [9].

Notably, in studies to date, the percussion and inversion treatment has been administered either in clinic or during inpatient admission, necessitating prolonged patient admission or increased follow-up regimes, which is costly. Our aim, therefore, was to evaluate the efficacy of patient-directed percussion and inversion therapy in the home setting on stone clearance rates post ESWL.

## 2. Materials and Methods

### 2.1. Trial Design

We undertook a prospective, randomised, controlled trial with clinician blinding. Patients with renal stones between 5 and 10 mm who underwent ESWL at a single institution between March and June 2023 were randomised in 1 : 1 allocation to either MPI (mechanical percussion and inversion therapy) or observation. All follow-up was conducted at the same institution and was completed in December 2023.

### 2.2. Participants

#### 2.2.1. Inclusion Criteria

Male and female patients aged 18 years or older, with CT proven single or multiple unilateral or bilateral renal stones measuring in total between 5 and 10 mm and visible on plain radiograph of kidney, ureter, and bladder, who were willing to undergo ESWL and able to tolerate inversion to >30 degrees were included.

#### 2.2.2. Exclusion Criteria

Patients were excluded if they had anatomical abnormalities including horseshoe kidney, fragments in a calyceal diverticulum, infundibular stenosis, pelvi-ureteric junction obstruction, or a ureteric stricture. All those with contraindications to ESWL including abdominal aortic aneurysm, anticoagulant, or antiplatelet treatment unable to be safely stopped peri-ESWL were also excluded. Further excluded from the study were those with medical conditions which made inversion dangerous including morbid obesity, uncontrolled hypertension, previous cerebrovascular accident, significant coronary artery disease, or symptomatic gastro-oesophageal reflux disease.

Patients with previous ipsilateral stone treatment within 3 years were not enrolled, and those with radiolucent calculi including uric acid stones were also excluded. No patients had a ureteric stent as part of this study.

### 2.3. Interventions

ESWL was carried out using a Dornier Delta® 3 lithotripter machine (Dornier MedTech, Wessling, Germany). Only one kidney was treated as part of the study even if patients had a contralateral renal stone. All patients were treated under general anaesthesia in theatre as is the practice of this centre. Patients were treated at a supine position, with stone localisation using fluoroscopy or with ultrasound when required. The shockwave frequency was 100 shocks per minute with a maximum of 3000 shocks administered (or fewer if the stone was felt to have been totally fragmented). At the end of the procedure, patients were discharged and advised to drink 2-3 litres of water over the subsequent 3 days. No medical diuresis or alpha blocker therapy was administered, and no specific analgesia was prescribed on discharge.

Patients randomised to the treatment arm were given written instructions on how to perform the percussion and inversion therapy (see appendix). Thirty minutes prior to commencing treatment, patients were asked to drink 2 glasses of water (300 mls). Following this, they were asked to place themselves in an inverted position either using an inclined surface or by leaning over a piece of furniture with some pillows to support the upper body. They were instructed using sample pictures (appendix) to have their upper body at a downward angle of at least 30 degrees, with an ideal angle of 45 degrees. They were then required to get a friend or family member to strike the back over the treated kidney with the palmar surface of an open hand, with diagrams of kidney location provided (appendix). Force of percussion was instructed to be akin to a firm massage; firm enough to feel the vibration impact through the body but not enough to cause pain or bruising. They were then asked to perform the percussion continuously for 10 minutes with short breaks if necessary. Therapy was advised to commence 2 days after ESWL treatment and to be repeated 3 times a week for 12 weeks until their follow-up appointment. Patients were given a follow-up phone call at 6 weeks to check adherence to percussion and address any questions by the urology clinical nurse, who was not involved in outcome adjudication.

### 2.4. Outcomes

The primary outcome of the study was to compare the stone clearance rate in the control arm versus the MPI arm after 12 weeks.

The secondary outcomes of the study were the difference in stone clearance rates between immediate versus delayed MPI and the difference in stone clearance rates with MPI in the lower pole of the kidney compared to other areas of the kidney.

### 2.5. Follow-up and Assessment

All patients had a noncontrasted CT of the abdomen and an X-ray of the kidney, ureters, and bladder prior to treatment. Follow-up consisted of an X-ray at 12 weeks post ESWL, at the time of their routine clinic follow-up. The stone-free status was defined as stone burden of ≤4 mm on plain X-ray, which are considered clinically insignificant residual fragments (CIRFs) in the literature [10–12]. X-rays were assessed by a clinician who was blinded to the study grouping.

Following the initial 12-week study period, patients with residual stone fragments were offered crossover to the MPI group and were again assessed with plain radiograph at twelve weeks.

The 12-week follow-up period was adopted to coincide with the standard follow-up period of patients undergoing ESWL at our centre, which is supported in the literature and allows time for spontaneous passage of fragments which can occur in up to two thirds of patients in the first three months [10, 11, 13].

### 2.6. Sample Size

Sample size requirement was calculated using a free access online calculator using estimates taken from previous studies [6, 8, 14]. Using an estimated stone-free rate of 50% in the MPI group, 20% in the observation group (i.e., 30% difference) with a type I error of 0.05 and type II error of 0.2, the estimated sample size was 32 in each arm (64 total). Allowing a further 10% for withdrawal gave a total sample size of 70 (35 in each group)

### 2.7. Data Collection and Storage

Data collected included patient demographics (age, sex, and BMI), stone characteristics (size, density, composition, previous treatments, skin-to-stone distance, and retreatment required), ESWL data (length treatment, number of shocks, total energy, and length of stay), and complications (haematuria, colic, haematoma, and steinstrasse). Data were collected on a standard proforma, before being entered into a secure electronic database after deidentification.

### 2.8. Randomisation and Blinding

Randomisation was performed using an online randomisation service (Sealed Envelope Ltd. 2015. Simple randomisation service (online) (accessed 15 Jun 2023)) and was performed by the Urology Clinical Nurse Consultant (CNC). Doctors involved in the study were blinded to treatment groups.

### 2.9. Ethical Consideration

Patients were counselled regarding the trial at the time of booking for their ESWL procedure. They were provided with study information before signing an informed consent form. Ethical approval was granted via Metro North HREC, Queensland, Australia, HREC no.: HREC/2022/QPCH/84961.

### 2.10. Statistical Analysis

Statistical analysis was performed using IBM SPSS Statistics version 23.0 (IMB Corp., Armonk, NY, USA). Descriptive analysis and frequency were used to report patient baseline characteristics. Independent *T*-test was used to compare age and stone size between cohorts. Chi squared analysis was used to compare stone-free rates overall, in the lower calyx and renal pelvis. Fisher's exact test was used to compare stone-free rates in the middle and upper calyces. 2-sided p values were reported with significance set at *p* < 0.05.

## 3. Results

**Figure 1:**
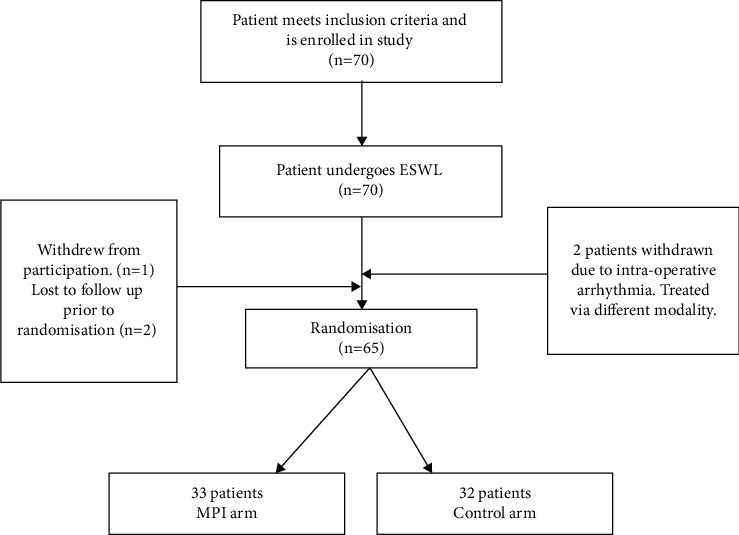
Patient selection flow chart.

70 patients met inclusion criteria and were enrolled in the study. 70 patients underwent ESWL. 2 patients had their procedure abandoned due to intraoperative arrythmia. They were removed and received a different treatment modality. 1 patient was elected to withdraw from the study postoperatively and a further 2 patients were uncontactable for randomisation and thus lost to the follow-up. This is depicted in Figure 1.

After standard practice ESWL, 33 patients were randomised to undergo MPI and 32 patients were randomised to the control group of ESWL alone (Table 1). All 65 patients attended their follow-up appointments. There was no difference in sex of patients in each group, with slight preponderance for males overall (35 males and 30 females). The average age of patients in the control group was 59 compared to 60 in the MPI group, which was not significant (*p*=0.80). There was no significant difference in stone size in the control group compared to the MPI group (*p*=0.43).

There was a statistically significant difference between the MPI group and the control group overall (*p*=0.089), with radiographic stone clearance in the MPI group of 87.8% (*n* = 29) compared to 59.4% (*n* = 19) in the control group.

With regards to lower calyceal stones, 43 patients were treated, with 24 randomised to MPI and 19 to the control group. The MPI group had a 91.7% (*n* = 22) radiographic clearance rate compared to 63.2% (*n* = 12) in the control group. This was statistically significant (*p*=0.022) (Table 2).

4 upper calyceal stones were treated; 2 randomised to each group, and all 4 patients had radiographic stone clearance and thus no treatment difference. 13 stones in the renal pelvis were treated, with 6 randomised to MPI and 7 to the control group. There was a 66.7% (*n* = 4) radiographic clearance rate in the MPI group compared to 71.4% (*n* = 5) in the control group, which was not significant (*p*=0.853). Similarly, there was no significant difference seen between the MPI and control groups regarding stones in middle calyces, with 5 stones treated; 4 in the control group showed 50% clearance rate and only 1 in the MPI which was cleared (Table 2).

Regarding delayed percussion, 10 patients from the control group who had residual fragments at 3 months were transferred to the MPI group. Of these, 60% (*n* = 6) had stone clearance at their further 3-month follow-up and 40% (*n* = 4) still had remaining fragments. 2 out of 3 patients from the primary MPI group who had a further 3 months of percussion passed their fragments, with 1 having a remaining stone burden. Thus, delayed percussion did not have a statistically significant impact on stone clearance (*p*=0.835).

Compliance was assessed via a study questionnaire. In the MPI group, the average number of sessions performed per week was 3.0, and the most common treatment duration was 6–10 minutes. 30% of the patients (*n* = 10) experienced some pain during MPI treatment, but no patients stopped treatment because of it. 24% of the patients (*n* = 8) experienced some haematuria during the MPI period, but this was similar to the control group (22%, *n* = 7). UTI/sepsis was infrequent and similar between the two cohorts (*n* = 1 for both groups), and there was no significant difference between steinstrasse in the MPI group (6%, *n* = 2) and the control group (3%, *n* = 1) (*p*=0.20) as seen in Table 3.

## 4. Discussion

Our study has shown that patient driven mechanical percussion and inversion therapy administered in a home setting can significantly affect stone-free rates after ESWL. Our clearance rates of 87.9% in the MPI arm for stones ≤10 mm anywhere in the kidney solidifies ESWL and postoperative MPI as a strong method of treatment for renal stones against the new wave of modern retrograde intrarenal surgery (RIRS).

MPI increased the passage rate of stones in the lower pole after ESWL by 28.5%. This encouraging result gives clinicians an adjunctive tool in managing the complexity of treating lower pole stones; an area where ESWL alone often has poor success (63% in control arm) and access endoscopically is difficult and often results in retained fragments.

It appears that immediate MPI therapy after ESWL is more useful than performing MPI in a delayed fashion. This may relate to increased stability of fragments over time, but further research is required to elucidate reasons for this. Furthermore, the relationship between primary versus delayed MPI was not investigated directly in this study as all patients were offered secondary MPI given chance of benefit.

Several studies have shown MPI therapy post ESWL to be effective in stone clearance, [6–8, 14, 15] but to our knowledge, this is the first study examining its use via self-directed therapy in a home setting. Our overall clearance rate appears comparable to that described by Ahmed et al. in 2015 [16], who performed ESWL in Trendelenburg position combined with intravenous frusemide and showed a stone-free rate of 90.9% for stones less than 10 mm, and Leong et al. [14] before them, who used intraoperative inversion alone and reported a 79.5% stone-free rate for similar sized stones. Self-directed therapy at home may reduce cost per treatment as well as frequency of follow-up.

More recent studies have begun to examine the type of instrument used to deliver percussion. The external physical vibration Lithecbole (EPVL) has shown promising results in improving stone-free rates both after ESWL as well as after RIRS (retrograde intrarenal surgery) [17–21]. Once again, our study has shown comparative results in increasing the stone-free rate anywhere in the kidney (28.4% in our study compared to 30.4% demonstrated by Wu et al. in 2017 [19]) and also in the lower calyces (28.5% in our study compared to 27.9% in the study by Long et al. in 2016 [17]). Further research is warranted to examine the difference between self-directed therapy and the use of physical devices such as the EPVL, both after ESWL as well as RIRS.

The major limitation of this study relates to adherence to therapy and standardisation of treatment that is self-driven without clinical supervision. The compliance rate in our study was fair, with the average treatment per week being equal to what was instructed. Despite treatment duration being shorter than that recommended, the results have still shown benefit in stone clearance rates. The nature of a trial setting, including the phone call during the follow-up period, likely led to increased adherence, which may not be replicable with larger numbers or outside a trial setting. Perhaps, this study can act as a platform to develop a standardised home treatment protocol which can be researched in the future.

A further limitation of the study was our use of X-ray in the follow-up of patients rather than noncontrast CT. This has likely resulted in undetected small residual fragments and thus a higher stone-free rate than if CT was used. We hope, however, that the uniformity of X-ray use between the MPI group and the control group has not affected the primary outcome of the study.

In conclusion, MPI therapy provides a promising solution to the inherent difficulty of treatment for lower pole stones as well as retained fragments post ESWL. Our study has shown that MPI therapy can be administered in a home setting with good efficacy, thus reducing the financial and resource burden of additional hospital-based treatments on both patient and health care systems. Further research is required to develop standardised home treatment protocols.

## Figures and Tables

**Figure 2:**
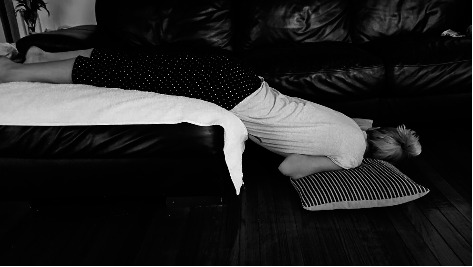
Suggested patient inversion position.

**Figure 3:**
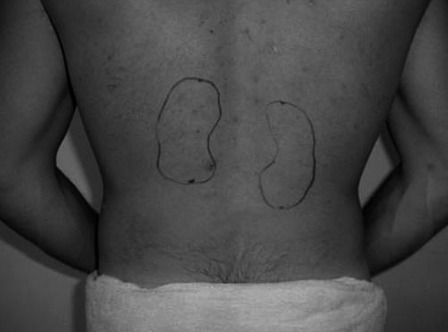
Suggested location for kidney percussion.

**Table 1:** Patient baseline characteristics.

	*N*	Mean age (y)	Sex		BMI (kg/m^2^)	Mean stone size (mm)	Location			
			Male	Female			LC	MC	UC	Pelvis
MPI	33	60.5	18	15	28.6	7.1	24	1	2	6
Control	32	59.1	17	15	29.2	7.4	19	4	2	7

MPI: mechanical percussion and inversion therapy; BMI: body mass index; LC: lower calyx; MC: middle calyx; UP: upper calyx.

**Table 2:** Results.

		*N*	Clearance at 3 months		*p* value
			*n*	%	
Overall	MPI	33	29	87.88	0.089
	Control	32	19	59.38	

LC	MPI	24	22	91.67	0.022
	Control	19	12	63.18	

Pelvis	MPI	6	4	66.67	0.853
	Control	7	5	71.43	

MC	MPI	1	1	100.00	1^*∗*^
	Control	4	2	50.00	

UC	MPI	2	2	100.00	1^*∗*^
	Control	2	0	0.00	

Delayed	Delayed MPI	10	6	60.00	0.835
	Primary + delayed MPI	3	2	66.67	

LP: lower calyx; MC: middle calyx; UP: upper calyx; MPI: mechanical percussion and inversion therapy; ^*∗*^Fisher's exact test.

**Table 3:** Complications.

	Pain		Haematuria		UTI/sepsis		Steinstrasse	
	*N*	%	*N*	%	*N*	%	*N*	%
MPI	10	30	8	24	1	3	2	6
Control	7	27	7	22	1	4	1	3

UTI: urinary tract infection.

## Data Availability

The data used to support the findings of this study are available on request from the corresponding author. Refined data are included within the article.

## Appendix

### A. Example of the Patient Instruction Sheet

How do I perform mechanical percussion and inversion therapy?

First, you will need to drink 2 glasses (300 mL) of water approximately 30 minutes before beginning your treatment.

Now you will need to place yourself in an inverted position. You may do this with an inclined surface or by leaning over a piece of furniture with some pillows to support your upper body. These are illustrated in the adjacent picture. No matter which method you choose, it is very important that you are well supported, comfortable, and not likely to fall.

You should aim to have your upper body on a downward angle of at least 30 degrees, with the ideal at 45 degrees. This means your shoulders should be the same distance below your hips as they are separated along the floor. Below is an example of positioning for MPI (Figure 2).

I am inverted, what do I do next?

Now you need to perform the mechanical percussion. You will need a friend or family member to assist you with this. With an open hand, the person assisting you should strike your back over the kidney which had the stone treated. The kidneys are located on either side of the middle of your back, under the last few ribs. The picture below should give you a guide to the correct location.

How firm should the percussion be?

The amount of force required is similar to that of a firm massage. The strike should be firm enough that you can feel a vibration through your body, though not so firm that it causes pain or bruising. If you get either of these, stop immediately and contact either the urology clinical nurse or urology registrar at the Prince Charles Hospital (Figure 3).

How many treatments should I do?

We ask that you perform the treatment three times per week for 10 minutes each session. We will ask you to fill out a questionnaire later on how many treatments you actually performed. The treatment will continue until the end of the study period of 12 weeks or until you are shown to be stone free at review.

What if I have any questions?

You can contact the urology nurse on *∗∗∗∗∗∗∗∗∗∗*.
